# Internet Use Amplifying Older Adults’ Existing Social Environments and Self-Perception of Aging

**DOI:** 10.1093/geroni/igaf122.2806

**Published:** 2025-12-31

**Authors:** Joonhyeog Park, Yanjun Dong, Zexi Zhou, Kun Wang

**Affiliations:** University of Pennsylvania, Philadelphia, Pennsylvania, United States; SUNY at Albany, Albany, New York, United States; The University of Texas at Austin, Austin, Texas, United States; University of Alabama at Birmingham, Birmingham, Alabama, United States

## Abstract

Negative social support, characterized by strained or harmful interactions, adversely affects older adults’ self-perceptions of aging (SPA). While digital engagement, such as Internet use, has the potential to expand social networks and enhance social support, it remains unclear whether it mitigates or exacerbates the detrimental effects of negative social support on SPA. This study addresses this gap by examining how Internet use moderates the relationship between positive/negative social support and positive/negative SPA. Data were drawn from the Health and Retirement Study (HRS): 2008/2010 (T1), 2012/2014 (T2), and 2016/2018 (T3) (n = 9,304, aged 65+). Random-effect panel regressions with interactions between Internet use and positive/negative social support were conducted. The results revealed that Internet use exacerbates the detrimental effects of negative social support on negative SPA (B = .11, p < .01) but does not significantly affect positive SPA. Conversely, Internet use enhances the benefits of positive social support on positive SPA (B = .07, p < .05) but has no significant effect on negative SPA. These findings suggest that Internet use amplifies the impact of existing social environments: it strengthens the benefits of positive social support while worsening the harm caused by negative social support. This highlights the need for targeted interventions tailored to older adults’ social contexts. In supportive environments, promoting digital engagement can strengthen positive age perceptions. In less supportive environments, interventions must combine digital engagement with efforts to reduce negative social interactions, ensuring the benefits of Internet use are accessible to all, regardless of social circumstances.

